# Genomic characterization of an Apennine heeling dog population: insights into diversity and conservation

**DOI:** 10.1016/j.vas.2026.100823

**Published:** 2026-08-17

**Authors:** Christian Persichilli, Roberta Ciampolini, Silvia Riggio, Marika Di Civita, Gabriele Senczuk, Filippo Biscarini, Chiara Zorzetto, Filippo Cendron, Salvatore Mastrangelo

**Affiliations:** a Department of Agricultural, Environmental and Food Sciences, University of Molise, Campobasso, Italy; b Department of Veterinary Sciences, University of Pisa, Pisa, Italy; c Department of Agricultural, Food and Forestry Sciences, University of Palermo, Palermo, Italy; d CNR, Institute of Agricultural Biology and Biotechnology (IBBA), Milan, Italy; e Apennine Heeling Dog Consortium, Pordenone, Italy; f Department of Agronomy, Food, Natural Resources, Animals and Environment, University of Padova, Legnaro, Italy

**Keywords:** local dog population, genomic data, population structure, genetic diversity, conservation

## Abstract

Over centuries, dogs have accompanied human populations during seasonal livestock movements associated with transhumance, contributing to the development of specialized pastoral dog populations. This study focuses on a population of heeling dogs distributed across the Italian Apennines, here referred to as Apennine heeling dogs, traditionally selected by shepherds based on homogeneous morphological and behavioral traits. The aim of the present study was to provide a genome-wide characterization of this population and to investigate its genetic relationships with other shepherd and herding breeds. A total of 16 individuals with documented pastoral ancestry (AppHeelDog) and 6 morphologically similar dogs with uncertain ancestry (AppMorph) were genotyped using a medium-density SNP array and analyzed together with publicly available datasets. The results indicate that the Apennine heeling dog forms a genetically coherent and distinct cluster across all analyses. ADMIXTURE revealed a characteristic ancestry component largely absent in other breeds, while TreeMix indicated a predominantly tree-like evolutionary history with no evidence of recent gene flow. A genomic affinity with continental European herding breeds, particularly German and Belgian Shepherd lineages, was also observed. Despite moderate genetic diversity, the population exhibited substantial genomic inbreeding and a reduced effective population size. The AppMorph group showed partial genetic overlap with the core AppHeelDog population but also evidence of heterogeneity, highlighting the importance of integrating genomic and phenotypic data when defining population boundaries. Overall, this study provides the first genomic evidence supporting the distinctiveness of the Apennine heeling dog and establishes a foundation for its recognition and conservation.

## Highlights

• Genome-wide analyses identify a distinct Apennine heeling dog cluster.
• A unique ancestry component differentiates this population from others.
• Moderate diversity coexists with high genomic inbreeding and low Ne.
• Tree-like history with no recent gene flow supports long-term isolation.
• Genomics supports recognition and conservation of this local lineage.

## 1 Introduction

Since their domestication, dogs (*Canis lupus familiaris*) have played a fundamental role in human societies, supporting a wide range of activities including hunting, guarding, transport, and livestock management (Stafford, 2006). Among these functions, pastoralism has been one of the primary drivers of canine diversification, particularly in mountainous and marginal areas where traditional farming systems persisted for centuries (Hancock, 2014).

In Europe, the practice of transhumance has profoundly shaped both cultural landscapes and the development of specialized dog populations adapted to specific environmental and functional demands. Herding dogs, and in particular heeling dogs (i.e., dogs that control livestock by working in close contact and applying controlled nipping to the animals’ hindquarters), possess an innate combination of physical and cognitive traits that enable them to move livestock and maintain group cohesion. Through generations of functional selection, specific components of the predatory motor pattern, such as eye fixation, stalking, and chasing, have been enhanced, while the terminal phases of the sequence, including bite and kill, have been effectively inhibited (Jeong et al., 2025). Historically, selection of shepherd dogs was based predominantly on working performance rather than standardized morphological criteria. This process led to the emergence of locally adapted populations shaped by environmental constraints, livestock management systems, and the cultural transmission of breeding practices (Lord et al., 2016).

In the Italian Apennines (a mountain range extending along the Italian peninsula), transhumance historically connected vast geographical areas, from the Northern Apennines to the central Apennine massif. Within this context, shepherds relied on versatile and resilient dogs capable of herding and controlling livestock across long distances and rugged terrains. Unlike officially recognized breeds, many of these local dog populations have been maintained outside formal breeding programs and shaped primarily by functional selection, environmental pressures, and culturally transmitted breeding practices. Italy hosts several local canine populations that are currently undergoing processes of recognition and warrant conservation and valorization (Bigi et al., 2015; Cortellari et al., 2021; Bionda et al., 2022). Despite the decline of traditional pastoralism, some of these populations have persisted, often in reduced numbers and under limited breeding control, potentially exposing them to genetic drift and inbreeding.

The local population investigated in this study consists of heeling dogs traditionally reared by both sedentary and transhumant shepherds across the Northern and Central Apennines. These dogs, here referred to as *Apennine heeling dogs*, display a notable degree of morphological and behavioral homogeneity despite lacking formal recognition by the Italian Kennel Club (ENCI), and no official census or pedigree records are currently available. Phenotypically, they are characterized by a long, trotting conformation, a dolichocephalic head with a pronounced stop, erect ears, robust musculature, short or rough coat types with variable coloration, and the presence of rear dewclaws (Supplementary Fig. S1) (Persichilli et al., 2025). Behaviorally, they are valued for their responsiveness, endurance, and efficiency in close-contact livestock handling.

In recent years, genome-wide analysis has become a powerful tool for the investigation of the genetic structure, diversity, and evolutionary history of dog populations (Vonholdt et al., 2010; Lequarré, et al., 2011; Wayne and Vonholdt, 2012; Ostrander et al., 2017). These approaches are particularly informative for local or unrecognized populations, as they enable the identification of genetic uniqueness, admixture patterns, levels of diversity, and relationships with established breeds (Pertoldi et al., 2013; Shannon et al., 2015; Parker et al., 2017; Mastrangelo et al., 2018; Cortellari et al., 2023). Such studies contribute not only to the understanding of canine domestication and breed formation but also to the conservation of genetic resources linked to traditional agro-pastoral systems.

The aim of the present study was therefore to provide a comprehensive genome-wide characterization of the Apennine heeling dog population and to investigate its genetic relationships with other shepherd and herding breeds from neighboring regions and the broader international context. By integrating genomic data with historical information, this work seeks to clarify the genetic identity of this population and to support its potential recognition and conservation within the framework of European pastoral heritage.

## 2 Materials and Methods

No animals were sacrificed for the purposes of this study. Biological samples were obtained from non-invasive buccal swabs collected with the informed consent of the dog owners, in accordance with national regulations on animal welfare. Samples were collected using a commercial DNA testing kit (Embark Veterinary, Inc.).

### 2.1 Animals and Genotyping

Although the degree of relatedness among sampled dogs could not be formally assessed, individuals were selected from different flocks and geographical areas, based on information provided by owners and breeders, to maximize the genetic diversity represented within the population. The Apennine heeling dog dataset included two groups: (i) 16 individuals with documented pastoral ancestry (AppHeelDog), selected based on historical records and consistent phenotypic characteristics; and (ii) 6 dogs from the same geographical area displaying similar morphological features but with uncertain ancestry (AppMorph). The latter group was included to investigate potential genetic affinities with the recognized pastoral lineage.

Genomic DNA was extracted from biological samples following standard protocols. All samples were genotyped using the Embark™ SNP genotyping platform, which is based on the Illumina CanineHD™ BeadChip and provides high-density genome-wide coverage across the canine genome (∼230K SNPs).

The newly generated genotypes were merged with the LUPA consortium dataset, which includes a broad representation of dog breeds and populations (Vaysse et al., 2011) and further integrated with publicly available genotype data from 11 sheepdog breeds and 4 Italian breeds (Hayward et al., 2019). Two wild canid species from the LUPA dataset, the grey wolf (Canis lupus) and the coyote (Canis latrans), were included as outgroups (Supplementary Table S1).

### 2.2 Quality Control

Quality control was performed using PLINK v1.9 (Chang et al., 2015). Only autosomal SNPs shared among datasets were retained. SNPs with a call rate lower than 95% (–geno 0.05) and minor allele frequency (MAF) lower than 0.05 (–maf 0.05) were excluded. Individuals with more than 10% missing genotypes (–mind 0.1) were also removed. Hardy–Weinberg equilibrium (HWE) filtering was applied using a threshold of 1e-50 (–hwe 1e-50). After filtering, the final dataset consisted of 1,214 individuals from 67 breeds (including wild canid species) and 90,181 autosomal SNPs, which were used for subsequent analyses.

One AppHeelDog individual was excluded during the quality control procedure because it did not meet the established filtering criteria.

### 2.3 Genetic diversity indices for Apennine heeling dogs

Genetic diversity indices were estimated using PLINK v1.9, including observed (Ho) and expected (He) heterozygosity. Contemporary effective population size (cNe) was estimated using SNeP v1.11 (Barbato et al., 2015). Runs of homozygosity (ROH) were identified using a sliding-window approach in PLINK with the following parameters: (i) a sliding window size of 15 SNPs; (ii) a proportion of homozygous overlapping windows of 0.1; (iii) a minimum of 15 consecutive SNPs required to define a ROH; (iv) a minimum ROH length of 1 Mb; (v) a maximum gap of 1,000 kb between consecutive homozygous SNPs; (vi) a density of one SNP per 10 kb; and (vii) a maximum of one SNP with missing genotype and up to one heterozygous genotype were allowed in a ROH. The inbreeding coefficient based on ROH (F_ROH_) for each animal was calculated.

### 2.4 Population structure analyses among populations

Genetic differentiation among breeds was explored through multidimensional scaling (MDS) analysis of SNP genotyping data, based on pairwise identity-by-state (IBS) distances computed in PLINK v1.9 (Chang et al., 2015). The first two MDS components were plotted to visualize clustering patterns and genetic proximity among populations.

Population genetic structure was further investigated using ADMIXTURE (Alexander and Lange, 2011). This analysis was performed testing K values ranging from 1 to 22. The most likely number of clusters was determined based on the cross-validation (CV) error procedure implemented in ADMIXTURE. Graphical representations of ancestry proportions were generated using the membercoef.circos function in the R package BITE (Milanesi et al., 2017).

To reconstruct genome-wide relationships among populations and to investigate historical patterns of population splits and gene flow, a maximum likelihood phylogenetic tree was inferred using TREEMIX (Pickrell and Pritchard, 2012). For this analysis, genotypic data from two wild canid species, the Grey Wolf (*Canis lupus*) and the Coyote (*Canis latrans*), were included as outgroups. Migration events were modeled by allowing the number of migration events to vary from 1 to 5. The optimal number of migration edges (intended as statistical proxies for migration events) was determined using the optM function implemented in the R package OptM (Fitak, 2019), based on changes in the model likelihood.

All these analyses were initially conducted on the complete dataset (Supplementary Table S1) to assess the position of the Apennine heeling dog population within a broader genomic framework. Subsequently, a reduced dataset was constructed by selecting the most closely related breeds, identified based on the results of the initial population structure analyses (e.g., MDS and ADMIXTURE), to investigate in greater detail the genetic relationships between Apennine heeling dogs and these populations (Table 1).

**Table 1:** Breeds and populations included in the reduced dataset, with corresponding abbreviations and number of sampled individuals.

Breed	Abbreviation	N
*Apennine heeling dog*	*AppHeelDog*	15
	*App_Morph*	6
Australian Shepherd	ASh	20
Australian cattle dog	AUCTL	10
Belgian malinois	BeM	6
Belgian shepherd (groenendael)	BeSHP	5
Belgian Tervuren	BeT	21
Border Collie	BoC	30
Bearded collie	BRDCOL	4
Cane corso	CCORSO	9
Coyote	COY	16
English shepherd	ESh	4
German Shepherd	GSh	30
Italian greyhound	ItGRY	6
Spinone italiano	ItSPI	2
Neapolitan mastiff	NEAPMAS	6
Old English shepherd	OLDESh	30
Shetland shepherd	SHET	26
Wolf	Wlf	16

### 2.5 Network analysis

Finally, a network-based analysis was performed to investigate fine-scale population structure between AppHeelDog and AppMorph and other four breeds (Belgian malinois – BeM; Belgian shepherd (Groenendael) – BeSHP; Belgian Tervuren – BeT; and German Shepherd- GSh). These breeds were chosen based on the closest breeds from the Treemix and Admixture analyses. Network construction and individuals’ clustering were implemented using the R version of NetView v2.1.0.9000 (Neuditschko et al., 2012; Steinig et al., 2016). The method requires specification of the number of nearest neighbours (K-NN) retained for each individual. K-NN was set up from 5 to 30, to investigate the characteristics of both fine- and large-scale genetic structures.

## 3 Results

### 3.1 Genetic Diversity Indices

The genetic diversity indices reported in Table 2 indicate that the Apennine heeling dog population shows a moderate level of genetic variability. Observed and expected heterozygosity values were highly comparable (Ho = 0.311 ± 0.198; He = 0.303 ± 0.172). The genomic fixation index (F_IS_) was close to zero and slightly negative (−0.027 ± 0.243), indicating no substantial deviation from Hardy–Weinberg expectations at the population level. In contrast, the genomic inbreeding coefficient estimated from runs of homozygosity revealed a relatively high degree of autozygosity (F_ROH_ = 0.269 ± 0.095). Finally, the effective population size estimated at the 13th generation was 145, consistent with the observed levels of genomic inbreeding and indicative of a restricted breeding population.

**Table 2:** Genetic diversity indices for Apennine heeling dogs. Observed (HO) and expected (HE) heterozygosity, inbreeding coefficient (FIS), and relative standard deviations (SD) are shown. Moreover, inbreeding based on runs of homozygosity (FROH) and effective population size at 13th generation (Ne13) are also reported.

Ho ± S.D.	He ± S.D.	F_IS_ ± S.D.	F_ROH_ ± S.D.	Ne 13th
0.311±0.198	0.303±0.172	-0.027±0.243	0.269±0.095	145

### 3.2 Genetic Relationship and population structure

**Fig. 1:**
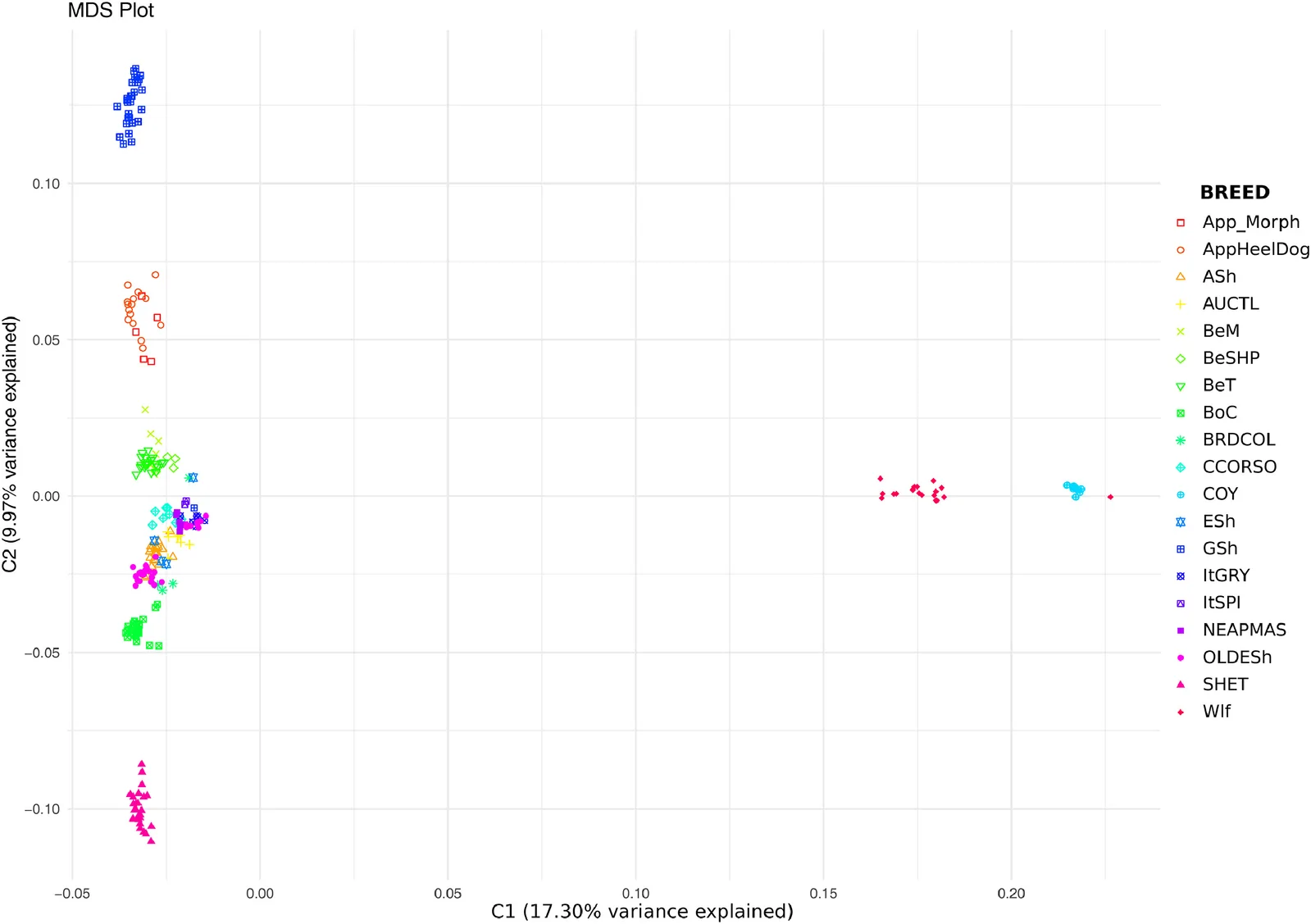
Genetic relationships inferred by multidimensional scaling (MDS) analysis among breeds and populations included in the reduced dataset. Breed acronyms are defined in Table 1.

The MDS analysis was performed on the full dataset, including a broader set of populations (N = 67), and provided the initial framework from which the reduced dataset shown in Fig. 1 was subsequently derived (Supplementary Fig. S2A and S2B). The first (C1) and second (C2) MDS coordinates explained 7.53% and 6.74% of the total genetic variance, respectively. At the individual level (Supplementary Fig. S2A), most populations clustered centrally, indicating limited genetic differentiation among the majority of breeds included in the full dataset. AppHeelDog and AppMorph samples were positioned in proximity and showed a strong overlap, forming a compact cluster within the central region of the plot. Several breeds in the full dataset were clearly separated from the main cluster, occupying peripheral positions along C1 and/or C2. The centroid-based MDS representation (Supplementary Fig. S2B) confirmed the individual-level patterns, with AppHeelDog and AppMorph centroids overlapping near the center of the genetic space, while centroids from more divergent breeds were further apart. In the reduced dataset (Fig. 1), genetic structuring became more evident, with C1 and C2 explaining 17.30% and 9.97% of the variance, respectively. Most populations clustered tightly on the left side of the plot, indicating relatively limited genetic differentiation along C1 and suggesting a broadly shared genetic background among these groups. AppHeelDog and AppMorph again clustered tightly together and were clearly separated from most other populations, primarily along the second axis, supporting the presence of a distinct and coherent genetic background. This shared clustering was primarily driven by separation along the C2 axis and was characterized by a compact and well-defined distribution of samples. In contrast, a small number of populations were clearly separated along the C1 axis and positioned on the far right of the plot, reflecting substantial genetic divergence from the remaining groups and showing minimal overlap with the main cluster.

**Fig. 2:**
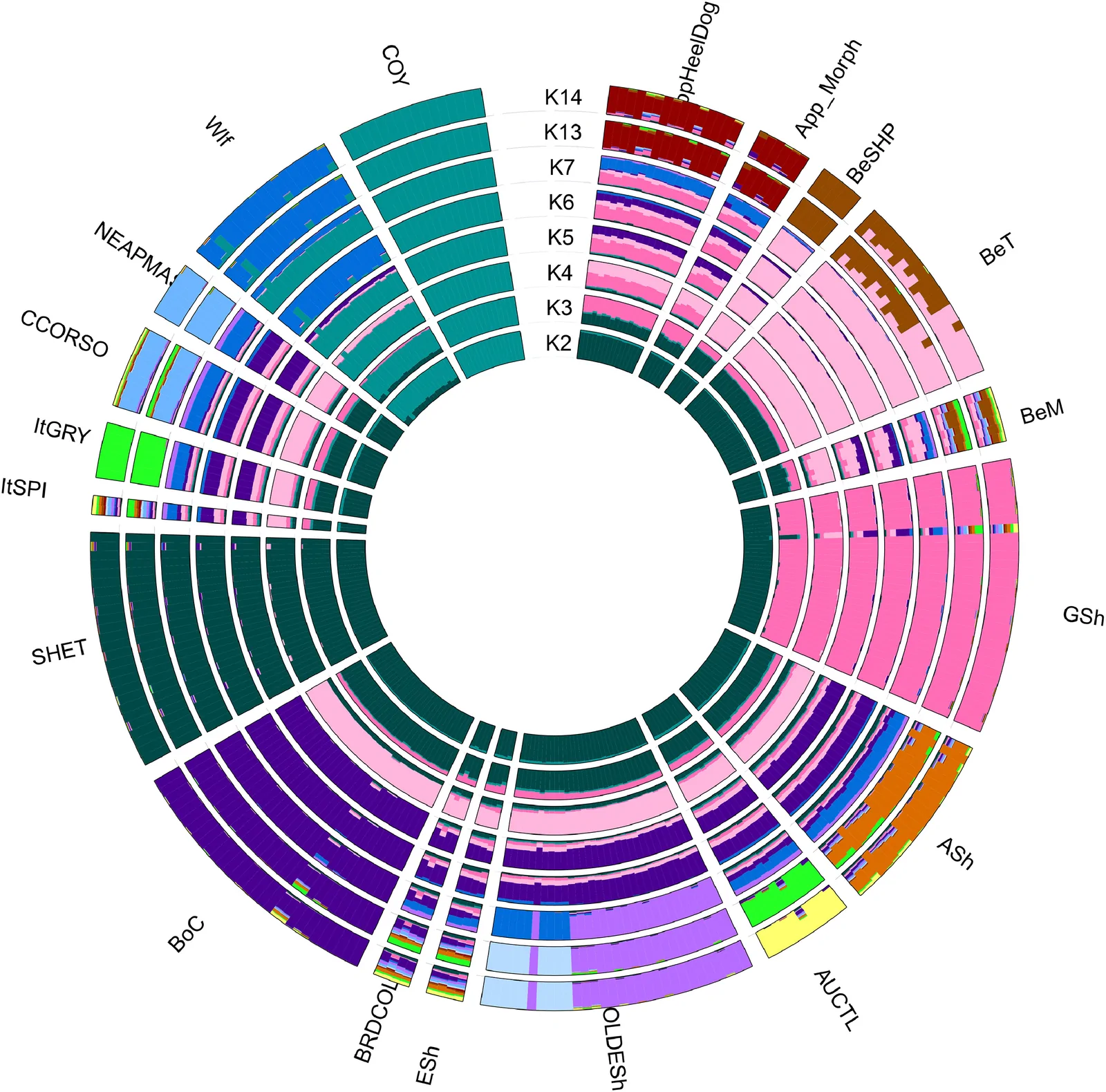
Model-based clustering of breeds and populations included in the reduced dataset for different K values. Breed acronyms are defined in Table 1.

ADMIXTURE analysis on the full dataset (Supplementary Fig. S3) showed the expected transition from broad-scale structure at low K values to finer ancestry resolution at higher K. At K2–K3, most populations were dominated by a limited number of major components, while outgroup populations (e.g., wolf and coyote) were clearly differentiated. With increasing K (K4–K7), several populations displayed homogeneous ancestry profiles (near single-component membership across individuals), whereas others retained visibly mixed profiles with multiple components within and among individuals. At K10 and especially K22, additional components emerged and further subdivided ancestry patterns within subsets of populations, increasing within-breed resolution and highlighting fine-scale substructure in groups that appeared more homogeneous at lower K. For higher resolution, Fig. 2 presents the ADMIXTURE outputs for a selected panel of populations (K = 2–7, 13, 14), making breed-level contrasts and within-breed heterogeneity easier to visualize. Several groups remained highly homogeneous across K (e.g., SHET consistently dominated by a single component; BoC similarly dominated by a distinct component), while other groups showed persistent multi-component composition and increasing fragmentation as K increased (e.g., CCORSO, BROCO, ESh, and BeM at higher K). GSh was consistently dominated by a single component across the displayed K values, whereas ASh showed a dominant component with additional fractions that became more apparent at intermediate and higher K. A key pattern highlighted in Fig. 2 concerns AppHeelDog and AppMorph. Across intermediate K (K4–K7), both groups exhibited structured ancestry profiles with a dominant component plus additional fractions, and their profiles became increasingly resolved as K increased. At K13–K14, AppHeelDog and AppMorph converged on a highly similar signature, being largely dominated by the same distinct high-K component (visually represented as the red cluster in the plot), with only minor residual contributions from other components. This high-K component was not prominent in most of the other populations shown in Fig. 2, indicating a clear differentiation of AppHeelDog/AppMorph relative to the remaining subset at these K values. In the same high-K solutions, BeSHP and BeT were characterised by a shared prominent component, while BeM retained a markedly heterogeneous multi-component pattern.

**Fig. 3:**
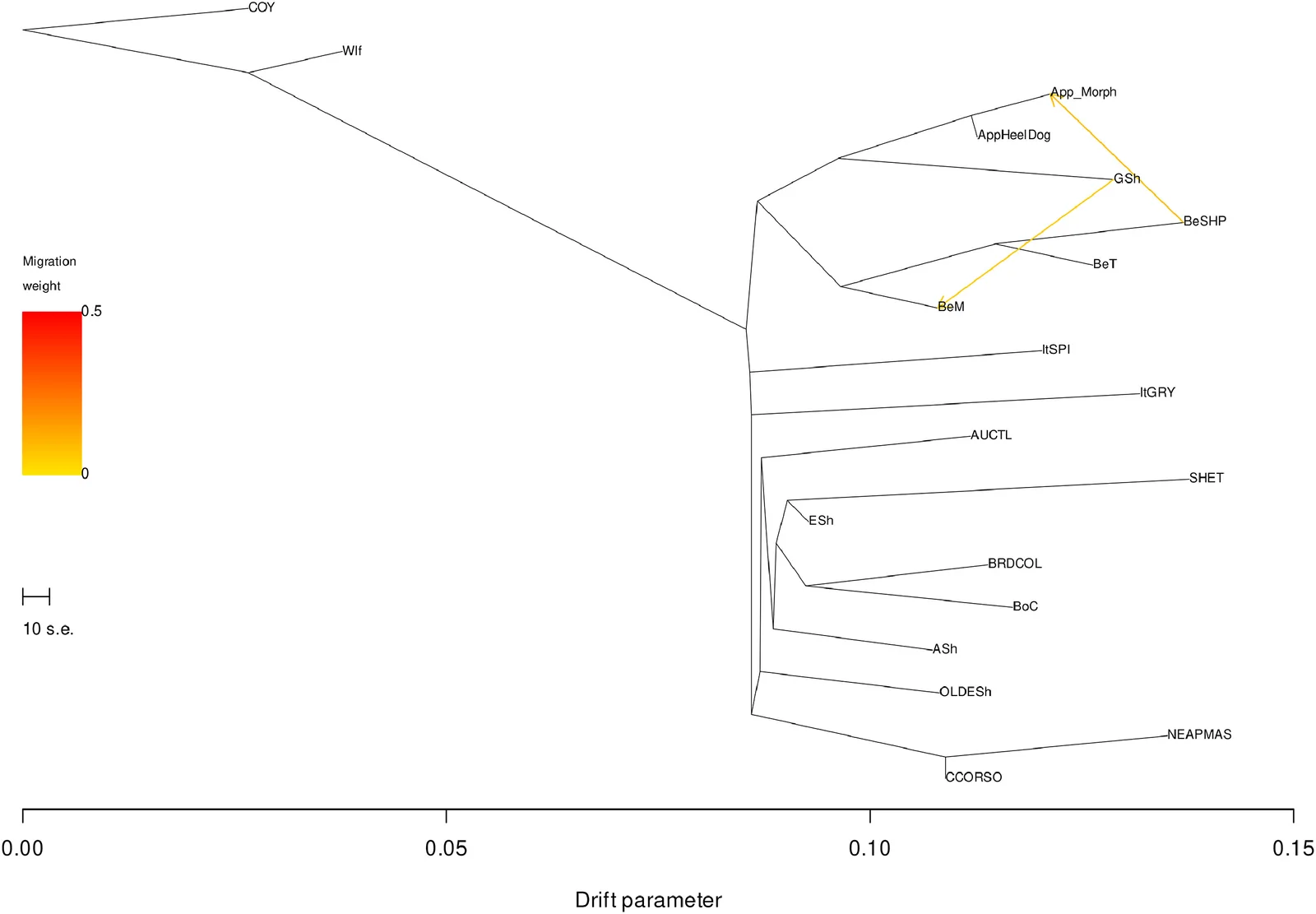
Phylogenetic network inferred with Treemix for the breeds and populations included in the reduced dataset. Breed acronyms are defined in Table 1.

TreeMix analysis on the full dataset (Supplementary Fig. S4) revealed a well-defined hierarchical structure, with populations distributed along branches of varying drift lengths. Several migration edges were inferred, primarily involving distantly related populations. AppHeelDog and AppMorph consistently clustered together, showing short drift distances relative to other groups. Notably, no migration edges directly connected to the Apennine cluster, suggesting an absence of detectable gene flow involving these populations. The analysis of the reduced dataset (Fig. 3) confirmed these patterns, with a simplified topology and fewer migration events. In the reduced dataset, migration events were characterized by lower migration weights and more localized connections between specific branches. AppHeelDog and AppMorph remained tightly grouped, and no migration edges were detected for this cluster, supporting a predominantly tree-like evolutionary history.

### 3.3 Network analysis

**Fig. 4:**
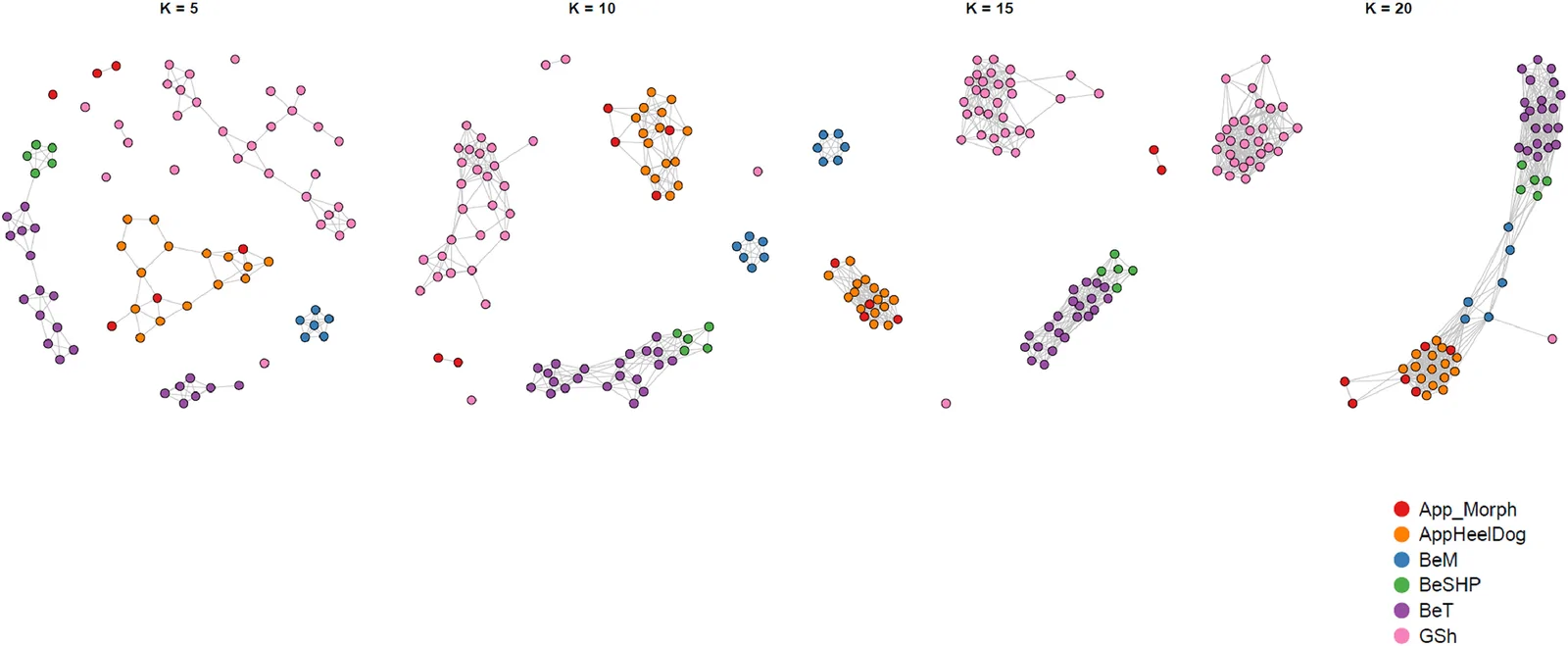
Mutual nearest-neighbour graphs obtained from NetView between *AppHeelDog and App_Morph* and other four breeds (Belgian malinois - BeM, Belgian shepherd - BeSHP, Belgian Tervuren - BeT, and German Shepherd- GSh) at k = 5, k = 10, k = 15, and k = 20.

Network analysis provided a fine-scale view of population structure confronting the studied population with the closest neighboring breeds according to the results of Treemix and ADMIXTURE analyses (Fig. 4). At lower K-NN values, the network was fragmented, with partially separated clusters. AppHeelDog formed a relatively cohesive group, while AppMorph individuals showed a more dispersed distribution. Belgian Malinois (BeM) formed a compact and clearly distinct component. Belgian Shepherd (BeSHP) and Belgian Tervuren (BeT) were organized into neighboring yet distinguishable groups. German Shepherd (GSh) individuals were distributed across partially connected subclusters. Increasing the K-NN value to 10 resulted in a marked rise in inter-individual connectivity, leading to a denser network structure. Cluster definition became more evident from K-NN = 15 onwards. The AppHeelDog cluster remained clearly distinguishable; four AppMorph individuals were positioned in close proximity to this cluster, whereas two samples consistently remained separate. Belgian breeds preserved their structured configuration, while GSh continued to form an independent and cohesive network. At K-NN = 20, intra-population connectivity was further consolidated, producing well-defined clusters. The AppHeelDog component remained compact, with AppMorph individuals integrated at its margins. Belgian breeds formed a continuous network structure, whereas GSh samples constituted a clearly separated cluster.

For K-NN values between 21 and 30 (Supplementary Fig. 5), network density progressively increased due to the addition of intra-population connections. However, the overall topology remained stable across this range, with no substantial changes in cluster configuration. Population-specific components remained clearly identifiable, and the relative positioning of AppHeelDog and AppMorph individuals was maintained. These additional network plots are provided to demonstrate the stability and robustness of the inferred population structure at higher K-NN values.

## 4 Discussion

The present study provides the first genome-wide characterization of the Apennine heeling dog population and supports the existence of a genetically coherent and distinctive pastoral dog group from the Italian Apennines. Genome-wide analyses have proven to be essential tools for resolving fine-scale population structure and identifying genetically distinct groups within domestic dogs, often revealing patterns that are not detectable using phenotypic or pedigree-based approaches (vonHoldt et al., 2010; Parker et al., 2017; Wiener et al., 2017). Several studies have demonstrated that dog populations shaped by specific functional roles, such as herding or livestock guarding, tend to form genetically coherent clusters reflecting both shared ancestry and long-term convergent selection for behavioral and ecological adaptation (Parker et al., 2004; Bigi et al., 2015; Shannon et al., 2015). Furthermore, local and regionally adapted dog populations have been increasingly recognized as important components of canine genetic diversity, often harboring unique genetic variants and evolutionary histories that are not represented in standardized breeds (Leroy, 2011; Dreger et al., 2016; Bionda et al., 2022). In this context, the identification of a distinct genetic cluster associated with the Apennine heeling dog supports the hypothesis that traditional pastoral systems have contributed to the maintenance of a structured and functionally specialized dog population in this geographic area.

### 4.1 Genetic diversity and inbreeding

The observed pattern of genetic diversity in the Apennine herding dog population reflects a balance between the maintenance of allelic variability and the accumulation of genomic inbreeding. The similarity between observed and expected heterozygosity, together with near-zero F_IS_ values suggests the absence of strong deviations from random mating. This pattern may indicate that, despite its relatively small size, the population has retained a measurable level of genetic variability (Mabunda et al., 2022). In contrast, the relatively high F_ROH_ values indicate substantial autozygosity, pointing to the accumulation of inbreeding over time. This pattern is consistent with long-term demographic processes, including population isolation, a limited number of founders, and repeated mating within a small and partially isolated breeding pool, rather than exclusively recent inbreeding events (Biscarini et al., 2026). Such genomic signatures are typical of small, functionally selected populations maintained outside formal breeding schemes. The relatively low effective population size further supports this interpretation, reflecting a limited pool of breeding individuals. Together, these findings suggest that the Apennine heeling dog population has experienced a demographic history characterized by relative isolation and limited gene flow, leading to the progressive accumulation of homozygous segments across the genome. An additional relevant aspect is the apparent contrast between moderate heterozygosity and high genomic inbreeding. This pattern is biologically meaningful, as it indicates that the population has retained a reservoir of allelic diversity while simultaneously accumulating long runs of homozygosity. This may reflect the coexistence of historical diversity with more recent demographic constraints, as well as mating practices driven primarily by functionality rather than genetic management. Similar patterns have been reported in other local or working dog populations maintained under traditional systems (Lampi et al., 2020; Proschowsky et al., 2025). Overall, these results indicate that, although genetic diversity has not collapsed, inbreeding has played a significant role in shaping the current genomic architecture of the Apennine heeling dog population, potentially increasing its vulnerability if not properly managed (Bigi et al., 2015).

### 4.2 Genetic relationships and population structure

Across all population structure analyses, Apennine heeling dogs consistently formed a compact and well-defined genomic cluster. This convergence across methods indicates a coherent genetic background and supports the interpretation of the population as a distinct genetic entity shaped by shared ancestry and functional selection (Ostrander et al., 2017; Cortellari et al., 2021; Bionda et al., 2023).

The differentiation of the Apennine heeling dog from most other analyzed herding and Italian populations was particularly evident in the reduced dataset, where a clear separation along the main axes of genetic variation was observed. ADMIXTURE analyses further reinforced this pattern, showing that at intermediate and higher K values the Apennine population was characterized by a distinctive ancestry component that was largely absent in most other populations. TreeMix analysis provided additional insights into the evolutionary relationships among populations. The inferred population tree consistently placed the Apennine heeling dog in close proximity to German Shepherd and Belgian Shepherd breeds, in agreement with the clustering observed in MDS and ADMIXTURE analyses. However, no significant migration edges involving the Apennine cluster were detected, suggesting that this population represents a relatively stable and distinct genetic lineage rather than the result of recent admixture events. This finding supports the hypothesis of a distinct genomic background and is consistent with the long-term maintenance of this dog population within relatively isolated pastoral systems (Boyko et al., 2014). The persistence of a recognizable ancestry signature despite the absence of formal breed recognition further highlights the role of traditional shepherd-driven selection in preserving population identity independently of kennel-club standardization.

The observed affinity with continental European herding breeds likely reflects either shared ancestry or convergent selection for similar functional traits associated with herding behavior (Parker et al., 2017). Given the historical role of transhumance in facilitating the movement of livestock, people, and working dogs across European mountain systems, such relationships are biologically plausible (Costello and Svensson, 2018). Nevertheless, the absence of detectable gene flow suggests that long-term isolation and local selection have played a major role in shaping the current genomic profile of the Apennine heeling dog (Talenti et al., 2018).

The AppMorph group represents an additional and particularly informative component of the analysis. These individuals showed substantial genomic overlap with the Apennine heeling dog cluster, indicating that part of the phenotypically identified local dog pool shares the same genetic background as the recognized pastoral lineage. However, the less cohesive placement of some AppMorph individuals in network analyses, together with signals of admixture observed in phylogenetic reconstruction, indicates a higher degree of heterogeneity within this group. Interestingly, one TreeMix configuration suggested a potential migration event from Belgian Shepherd (Groenendael) into the AppMorph group, a signal that was not observed for the core Apennine population. Although based on a limited number of individuals, this finding may reflect a more complex or heterogeneous origin of AppMorph dogs and further supports their distinction from the main Apennine heeling dog population (Talenti et al., 2018). Importantly, these results imply that phenotypic similarity alone may not be sufficient to accurately define population boundaries in local dog populations. The integration of genomic data is therefore essential to distinguish between core population members and individuals with admixed or uncertain ancestry, particularly in the context of conservation and potential breed recognition programs (Parker et al., 2017).

### 4.3 Conservation implications

Taken together, these findings have important conservation implications. The genomic distinctiveness of the Apennine heeling dog, combined with its strong association with transhumant pastoral systems, supports its value as both a biological resource and a component of regional biocultural heritage (Bigi et al., 2015; Cortellari et al., 2023). Local dog populations linked to traditional agro-pastoral systems represent unique reservoirs of genetic variation and functional adaptation. Their loss would imply not only the erosion of canine biodiversity but also the disappearance of cultural practices and knowledge systems developed over centuries (Shannon et al., 2015). In this context, the Apennine heeling dog emerges as a strong candidate for targeted conservation strategies. These should include systematic phenotypic and genealogical recording, the application of genomic tools to monitor inbreeding and inform mating decisions, and the development of structured breeding programs aimed at preserving both functional performance and genetic variability. More broadly, this study highlights the importance of integrating genomic approaches into conservation frameworks for local and still unrecognized dog populations, particularly those associated with traditional production systems undergoing rapid socio-economic change.

At the same time, the results should be interpreted as a first step toward a more comprehensive characterization of this population. Future studies including larger sample sizes, broader sampling of Italian rural dog populations, additional comparisons with traditional European herding breeds, and haplotype-based or demographic approaches will be essential to refine these interpretations.

## 5 Conclusions

This study provides the first genome-wide characterization of the Apennine heeling dog, demonstrating that this population constitutes a genetically identifiable and relatively homogeneous group within the broader context of European herding dogs. Despite detectable affinities with continental shepherd breeds, the Apennine heeling dog retains a distinct genomic signature shaped by long-term isolation and functional selection within transhumant pastoral systems. The combination of moderate genetic diversity, substantial genomic inbreeding, and a reduced effective population size highlight the impact of historical demographic processes and underscores the potential vulnerability of this population. These findings emphasize the need for informed management strategies aimed at preserving genetic variability while maintaining functional traits. Overall, this work provides a genomic baseline for the recognition, management, and long-term conservation of the Apennine heeling dog, supporting its valorization as part of the biocultural heritage of the Italian Apennines.

## Ethics declaration

This study was conducted in accordance with the following guidelines for animal welfare and/or reporting: No animals were sacrificed for the purposes of this study. Biological samples were obtained from non-invasive buccal swabs collected with the informed consent of the dog owners, in accordance with national regulations on animal welfare. Ethics committee approval was not required. No experimental studies were conducted on these animals. The authors confirm that they have followed EU standards for the protection of animals used for scientific purposes.

## Consent for publication

Not applicable.

## Ethical statement

No animals were sacrificed for the purposes of this study. Biological samples were obtained from non-invasive buccal swabs collected with the informed consent of the dog owners, in accordance with national regulations on animal welfare. Samples were collected using a commercial DNA testing kit (Embark Veterinary, Inc.), and the resulting genotypic data were voluntarily provided to the research team by the owners for use in this study. All data were handled in compliance with the European General Data Protection Regulation (GDPR), ensuring anonymity and confidentiality.

## Declaration of generative AI and AI-assisted technologies

During the preparation of the manuscript, generative AI and AI-assisted technologies were used exclusively for language editing. All content remains the responsibility of the authors.

## Funding

This research did not receive any specific grant from funding agencies in the public, commercial, or not-for-profit sectors

## CRediT authorship contribution statement

**Christian Persichilli:** Writing – original draft, Software, Formal analysis, Data curation. **Roberta Ciampolini:** Writing – review & editing, Visualization, Supervision, Investigation, Data curation, Conceptualization. **Silvia Riggio:** Writing – original draft, Software, Investigation, Formal analysis. **Marika Di Civita:** Writing – original draft, Methodology, Formal analysis, Data curation. **Gabriele Senczuk:** Writing – original draft, Investigation, Data curation. **Filippo Biscarini:** Writing – review & editing, Methodology, Investigation, Formal analysis, Data curation. **Chiara Zorzetto:** Writing – review & editing, Validation, Resources, Investigation, Conceptualization. **Filippo Cendron:** Writing – original draft, Investigation, Formal analysis, Data curation. **Salvatore Mastrangelo:** Writing – review & editing, Writing – original draft, Supervision, Methodology, Investigation, Data curation, Conceptualization.

## Declaration of competing interest

The authors declare that they have no known competing financial interests or personal relationships that could have appeared to influence the work reported in this paper.

## Data Availability

The data analysed in the current study are available from the corresponding author on reasonable request.

## Appendix Supplementary materials

Supplementary Figure S1 Example of the Apennine heeling dog Image, image 1 dummy alt text

Supplementary Figure S2. Genetic relationships among all 67 canid populations inferred by multidimensional scaling (MDS) analysis: (A) individual-level distribution; (B) population-level relationships based on average C1 and C2 coordinates. Breed acronyms are defined in Table S1. Image, application 2

Supplementary Figure S3. Model-based clustering of 67 canid populations at different numbers of inferred clusters (K). Breed acronyms are defined in Table S1. Image, application 3

Supplementary Figure S4. Phylogenetic network inferred with Treemix for the 67 canid populations. Breed acronyms are defined in Table S1. Image, application 4

Supplementary Figure S5. Fine- and large-scale network visualization of relatedness between *AppHeelDog and App_Morph* and other four breeds (Belgian malinois - BeM, Belgian shepherd - BeSHP, Belgian Tervuren - BeT, and German Shepherd- GSh), from k = 21 to k = 30. Image, application 5

Image, application 6
