# Supplementary material for: Genomic characterization of an Apennine heeling dog population: insights into diversity and conservation
Source: Vet Anim Sci. 2026 Aug 17;33:100823. doi: 10.1016/j.vas.2026.100823 (PMC13524634; doi:10.1016/j.vas.2026.100823)
Supplement: Supplementary file 1 [file mmc2.pdf]

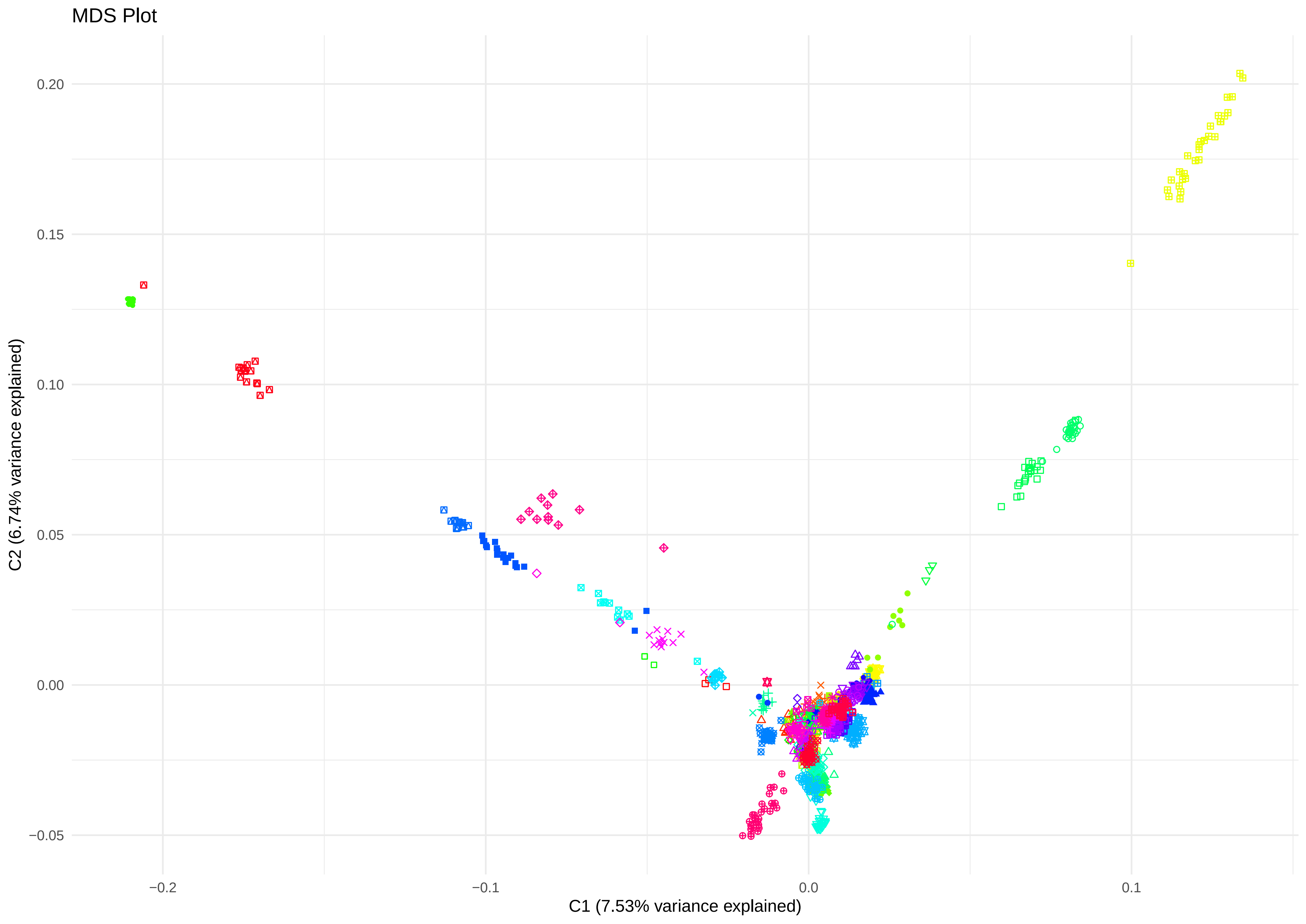
MDS Plot
C2 (6.74% variance explained)
C1 (7.53% variance explained)
0.20
0.15
0.10
0.05
0.00
−0.05
−0.2
−0.1
0.0
0.1

MDS Plot of Centroids

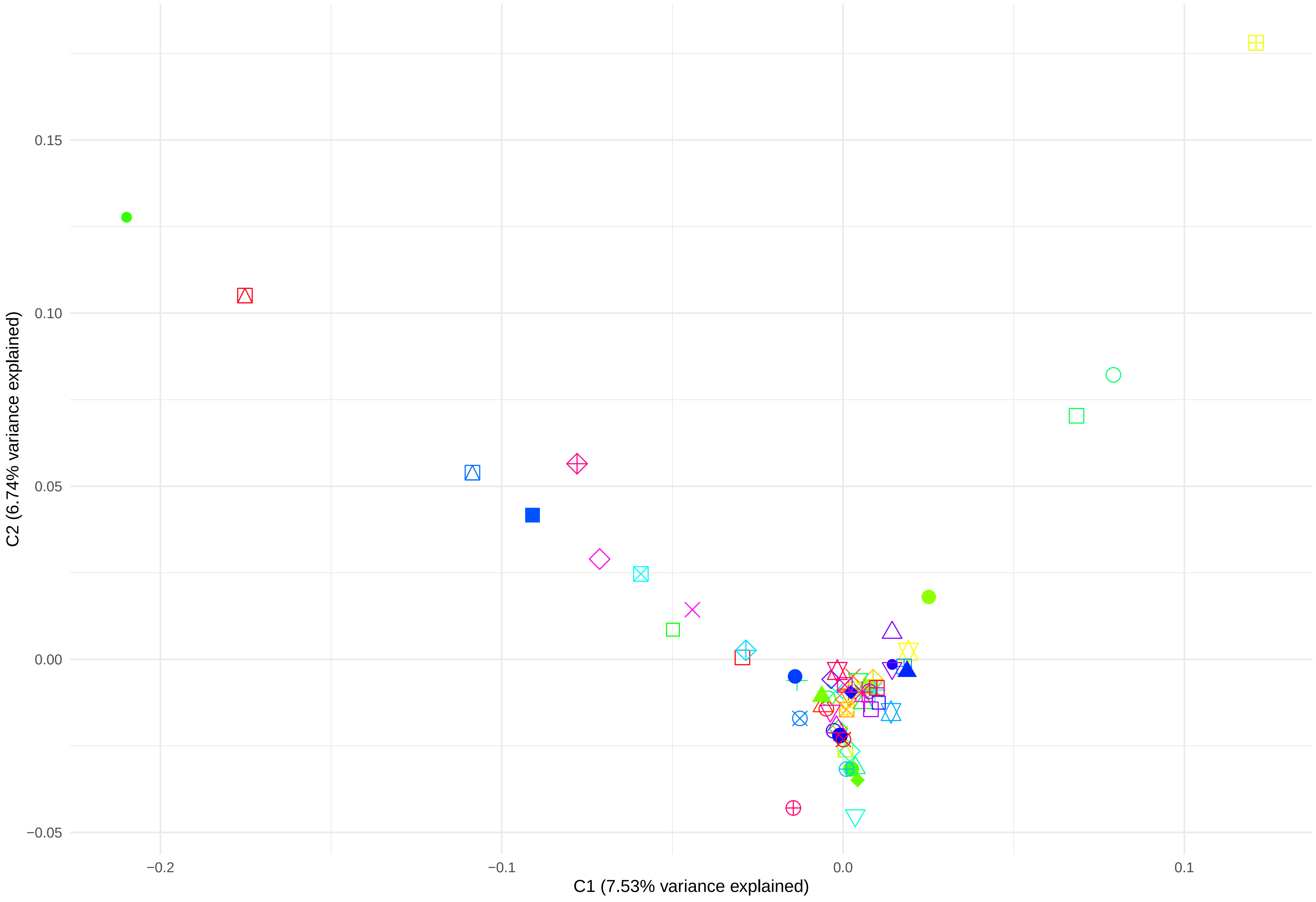

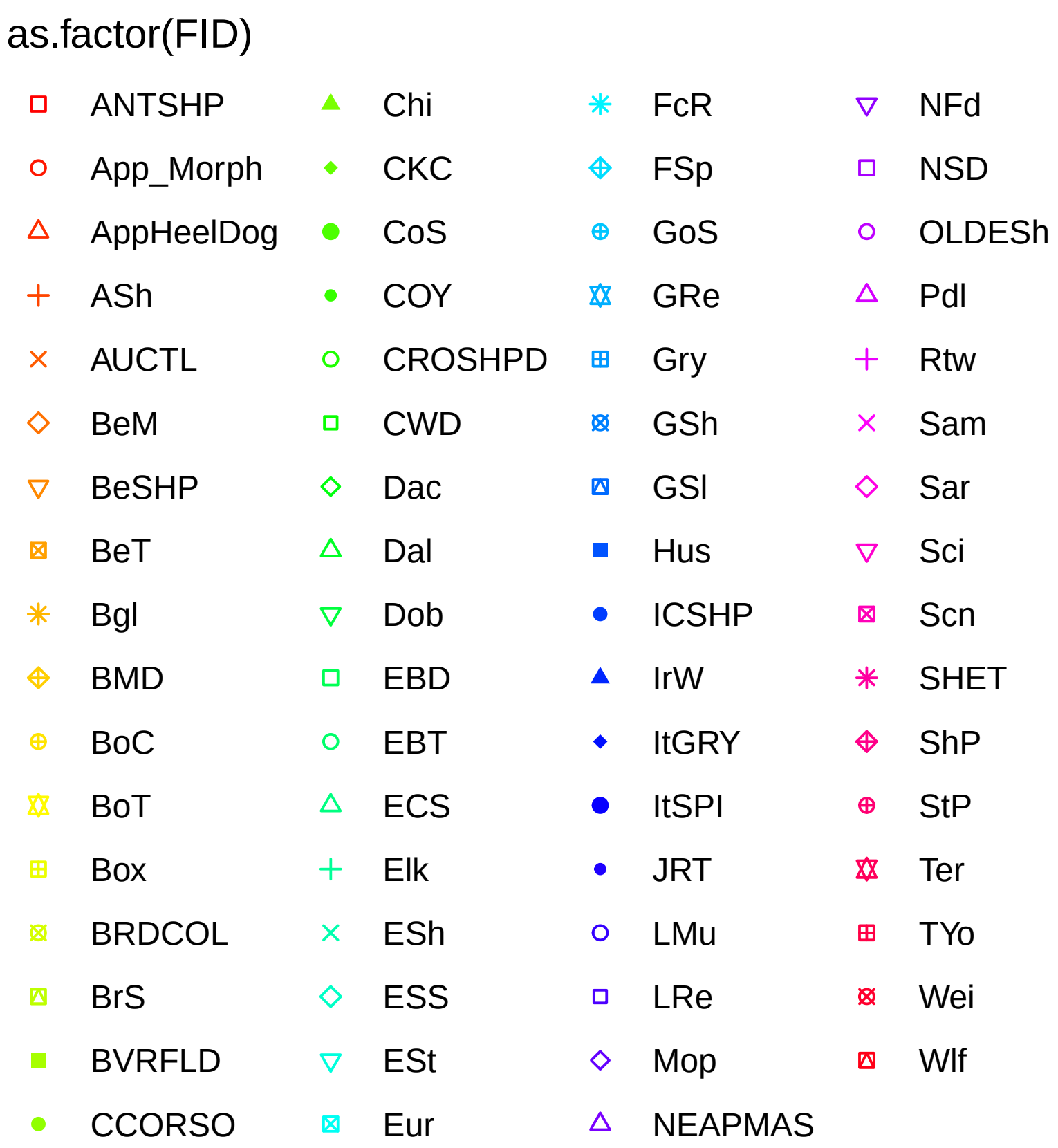
as.factor(FID)
ANTSHP
App_Morph
AppHeelDog
ASh
AUCTL
BeM
BeSHP
BeT
Bgl
BMD
BoC
BoT
Box
BRDCOL
BrS
BVRFLD
CCORSO
Chi
CKC
CoS
COY
CROSHPD
CWD
Dac
Dal
Dob
EBD
EBT
ECS
Elk
ESh
ESS
ESt
Eur
FcR
FSp
GoS
GRe
Gry
GSh
GSl
Hus
ICSHP
IrW
ItGRY
ItSPI
JRT
LMu
LRe
Mop
NEAPMAS
NFd
NSD
OLDESh
Pdl
Rtw
Sam
Sar
Sci
Scn
SHET
ShP
StP
Ter
TYo
Wei
Wlf
