# Supplementary material for: Genomic characterization of an Apennine heeling dog population: insights into diversity and conservation
Source: Vet Anim Sci. 2026 Aug 17;33:100823. doi: 10.1016/j.vas.2026.100823 (PMC13524634; doi:10.1016/j.vas.2026.100823)
Supplement: Supplementary file 3 [file mmc4.pdf]

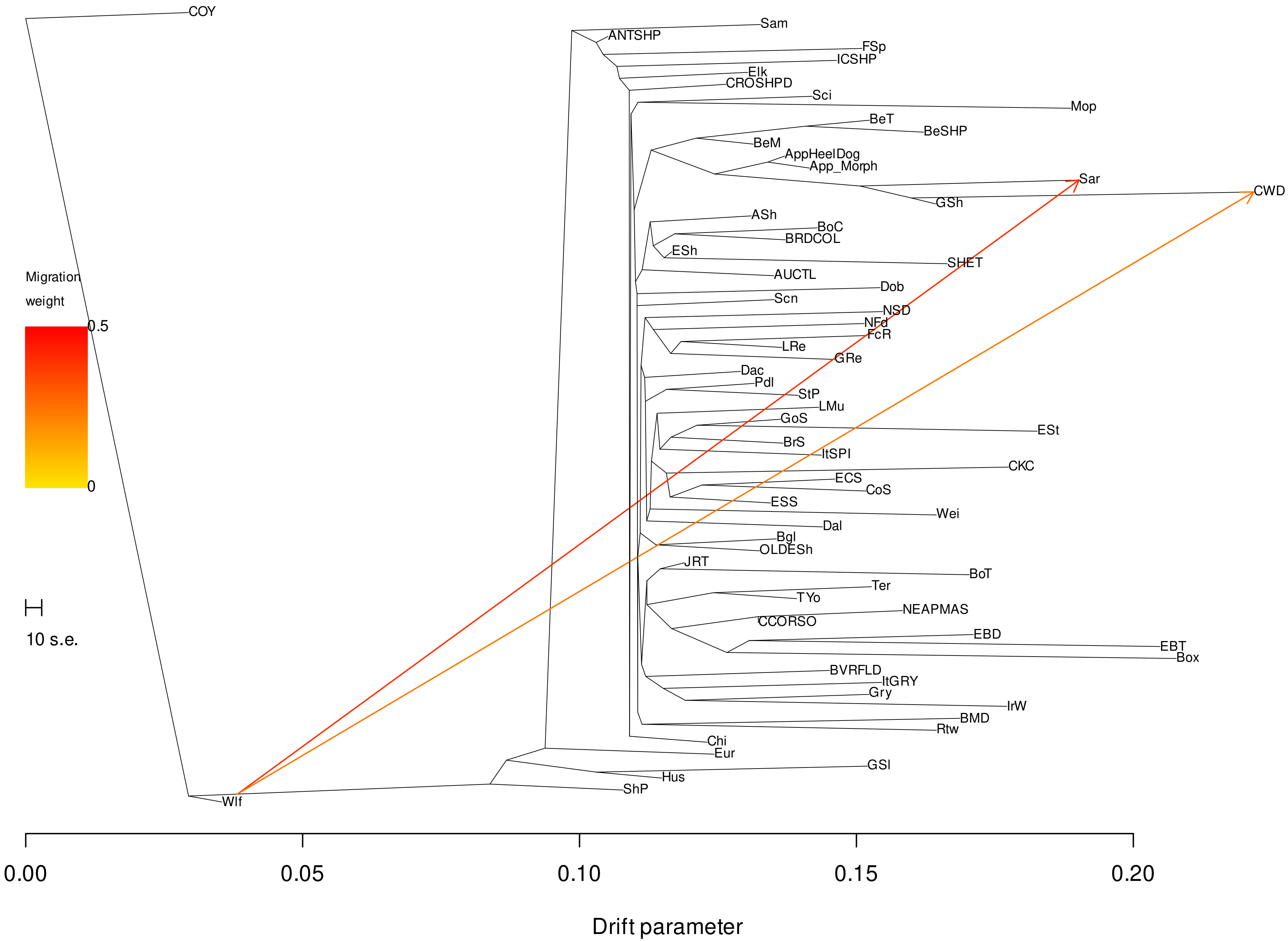

COY
Sam
ANTSHP
FSp
ICSHP
Elk
CROSHPD
Sci
Mop
BeT
BeSHP
BeM
AppHeelDog
App_Morph
Sar
CWD
GSh
ASh
BoC
BRDCOL
ESh
SHET
AUCTL
Dob
Scn
NSD
NFd
FcR
LRe
GRe
Dac
Pdl
StP
LMu
GoS
ESt
BrS
ItSPI
CKC
ECS
CoS
ESS
Wei
Dal
Bgl
OLDESh
JRT
BoT
Ter
TYo
NEAPMAS
CCORSO
EBD
EBT
Box
BVRFLD
ItGRY
Gry
IrW
BMD
Rtw
Chi
Eur
GSI
Hus
ShP
Wlf
Migration
weight
0.5
0
10 s.e.
0.00
0.05
0.10
0.15
0.20
Drift parameter
