# Supplementary material for: Genomic characterization of an Apennine heeling dog population: insights into diversity and conservation
Source: Vet Anim Sci. 2026 Aug 17;33:100823. doi: 10.1016/j.vas.2026.100823 (PMC13524634; doi:10.1016/j.vas.2026.100823)
Supplement: Supplementary file 4 [file mmc5.pdf]

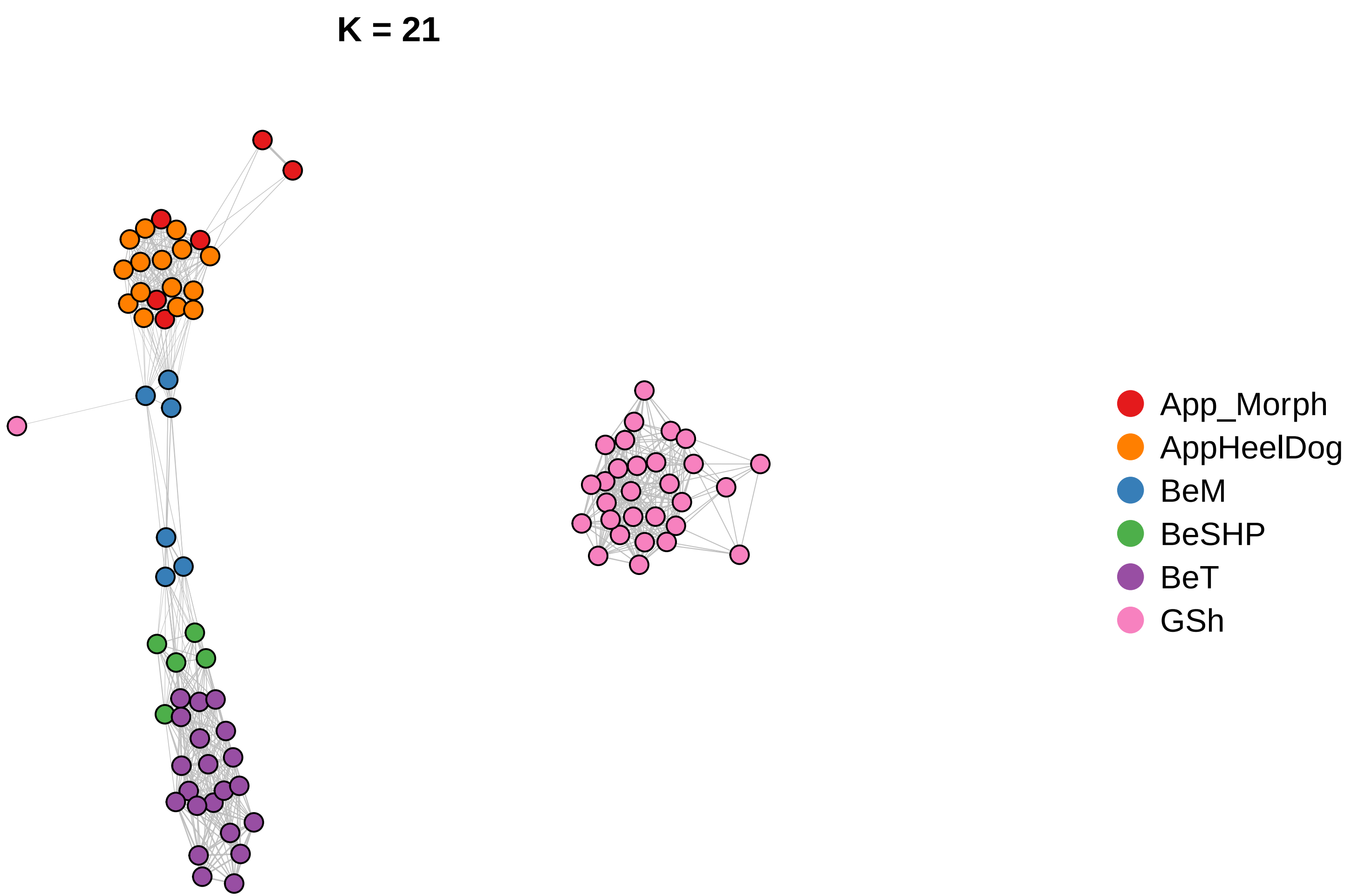
K = 21
App_Morph
AppHeelDog
BeM
BeSHP
BeT
GSh

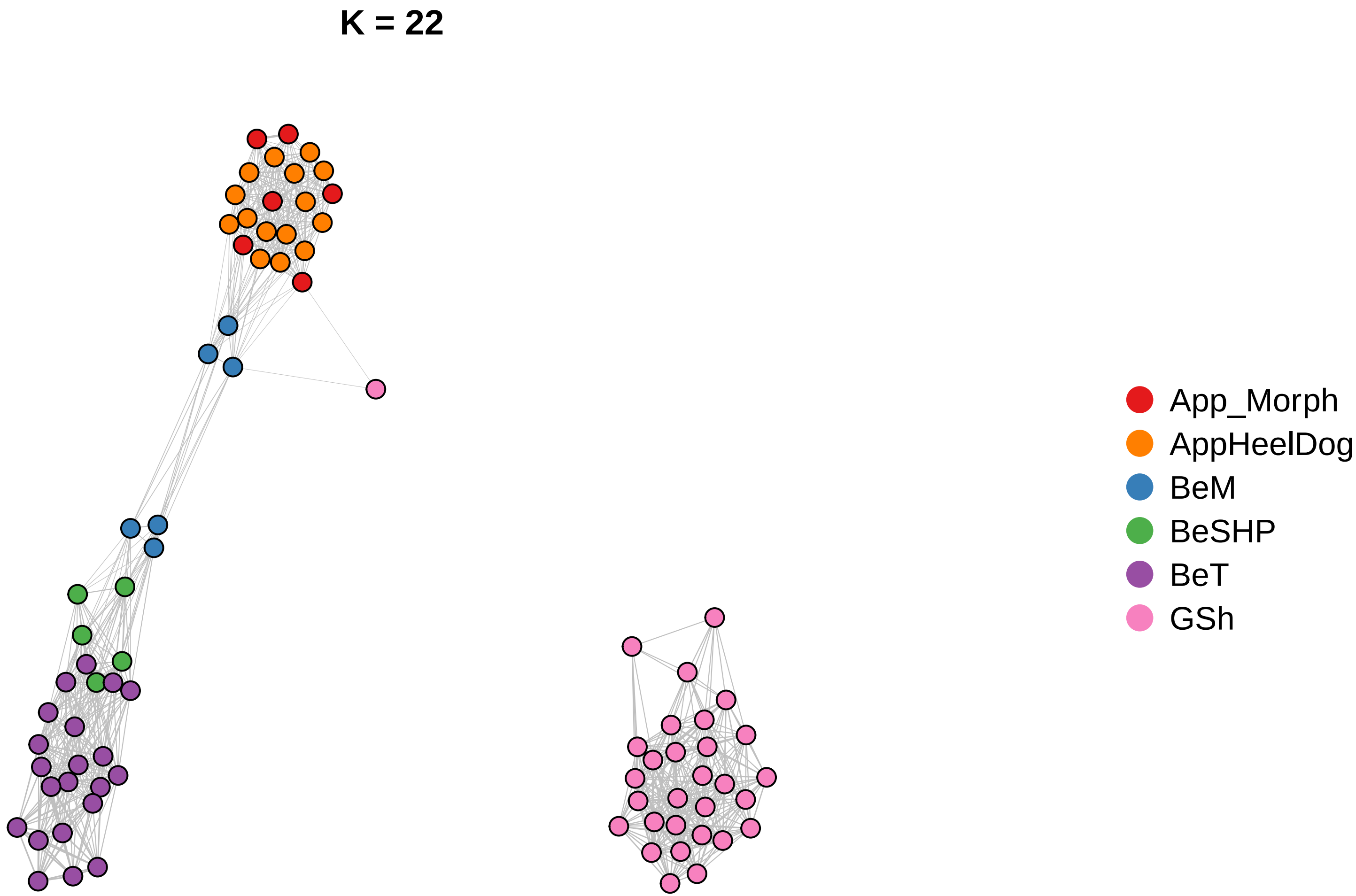
K = 22
App_Morph
AppHeelDog
BeM
BeSHP
BeT
GSh

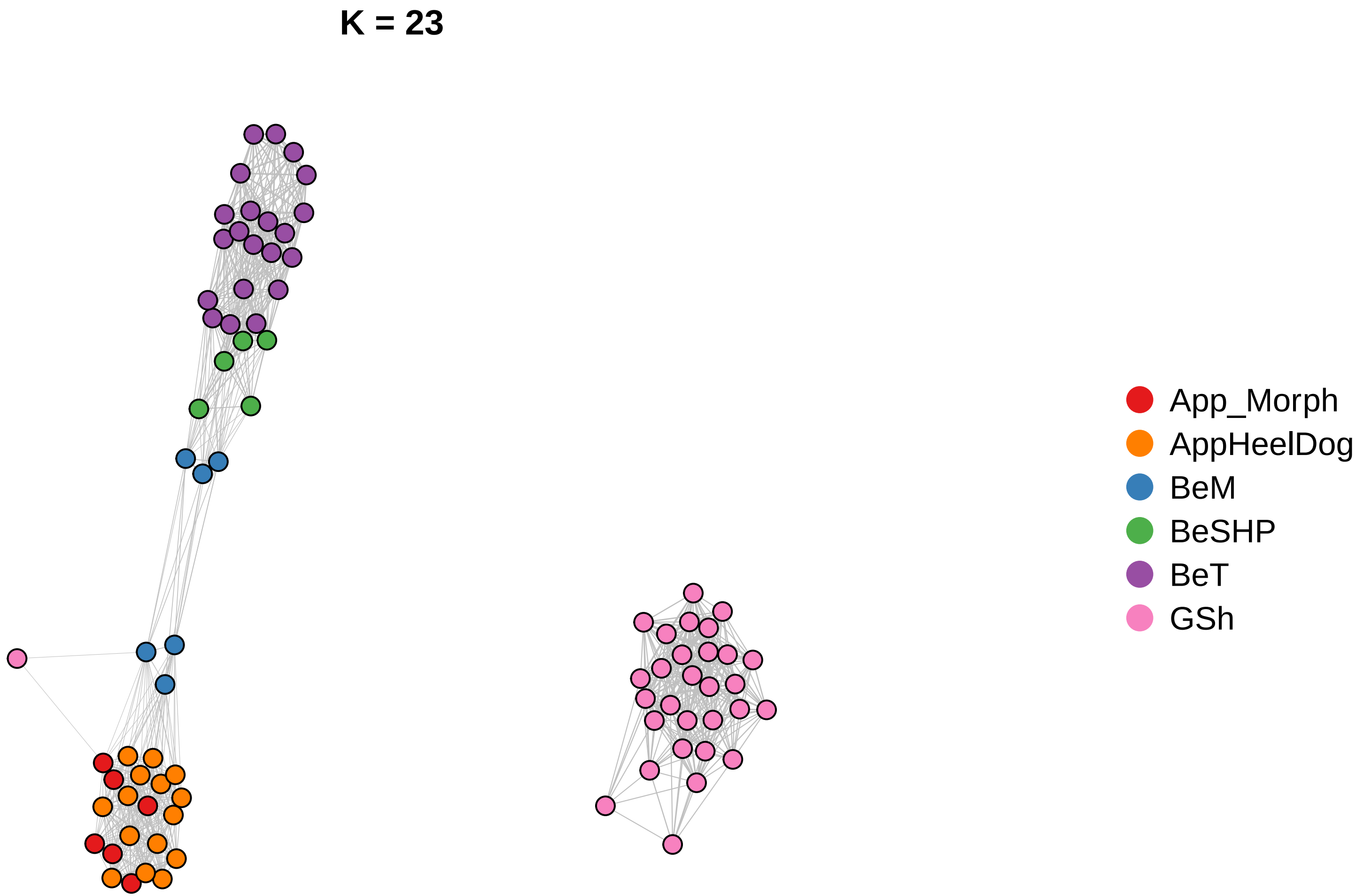
K = 23
App_Morph
AppHeelDog
BeM
BeSHP
BeT
GSh

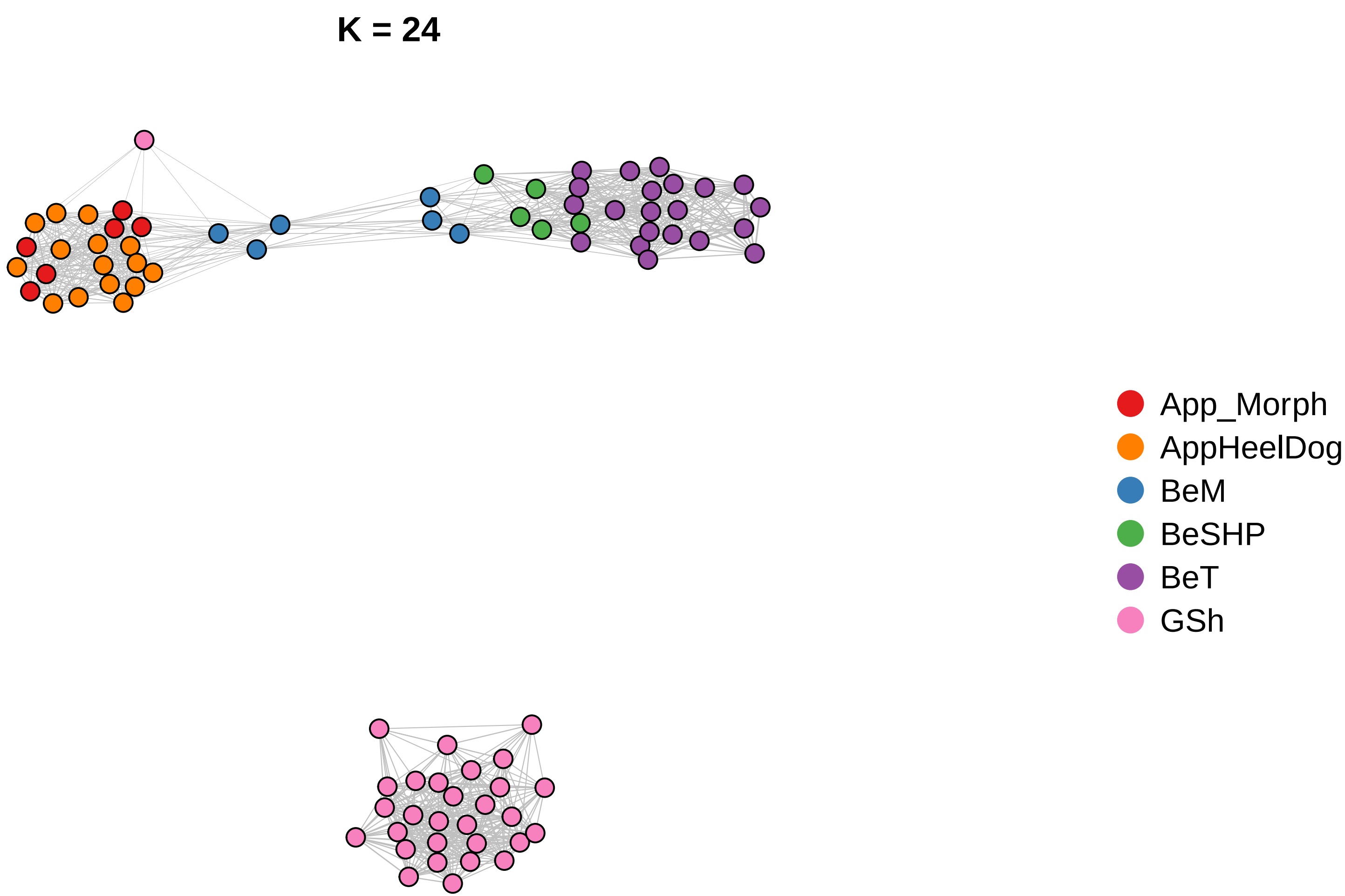
K = 24
App_Morph
AppHeelDog
BeM
BeSHP
BeT
GSh

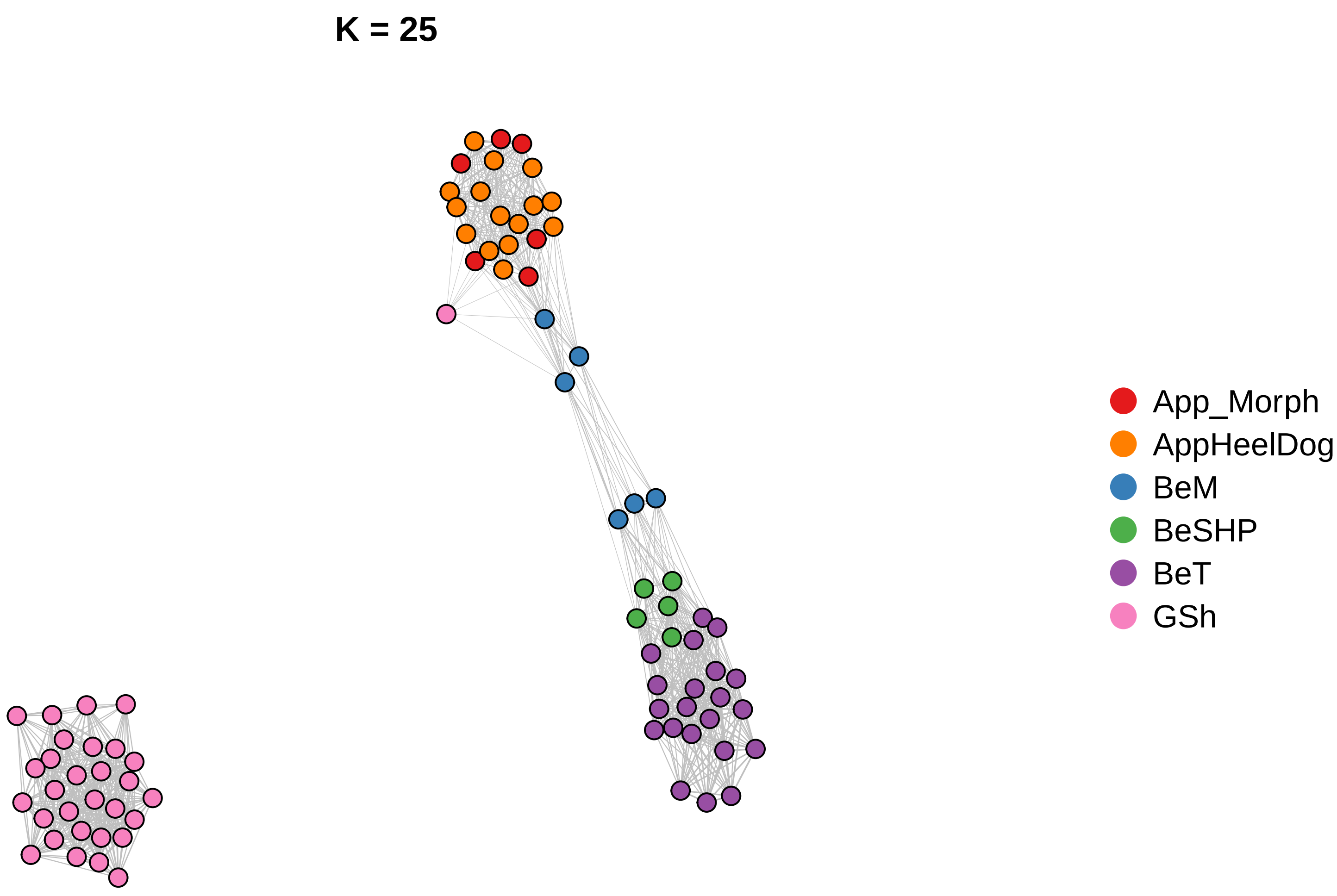
K = 25
App_Morph
AppHeelDog
BeM
BeSHP
BeT
GSh

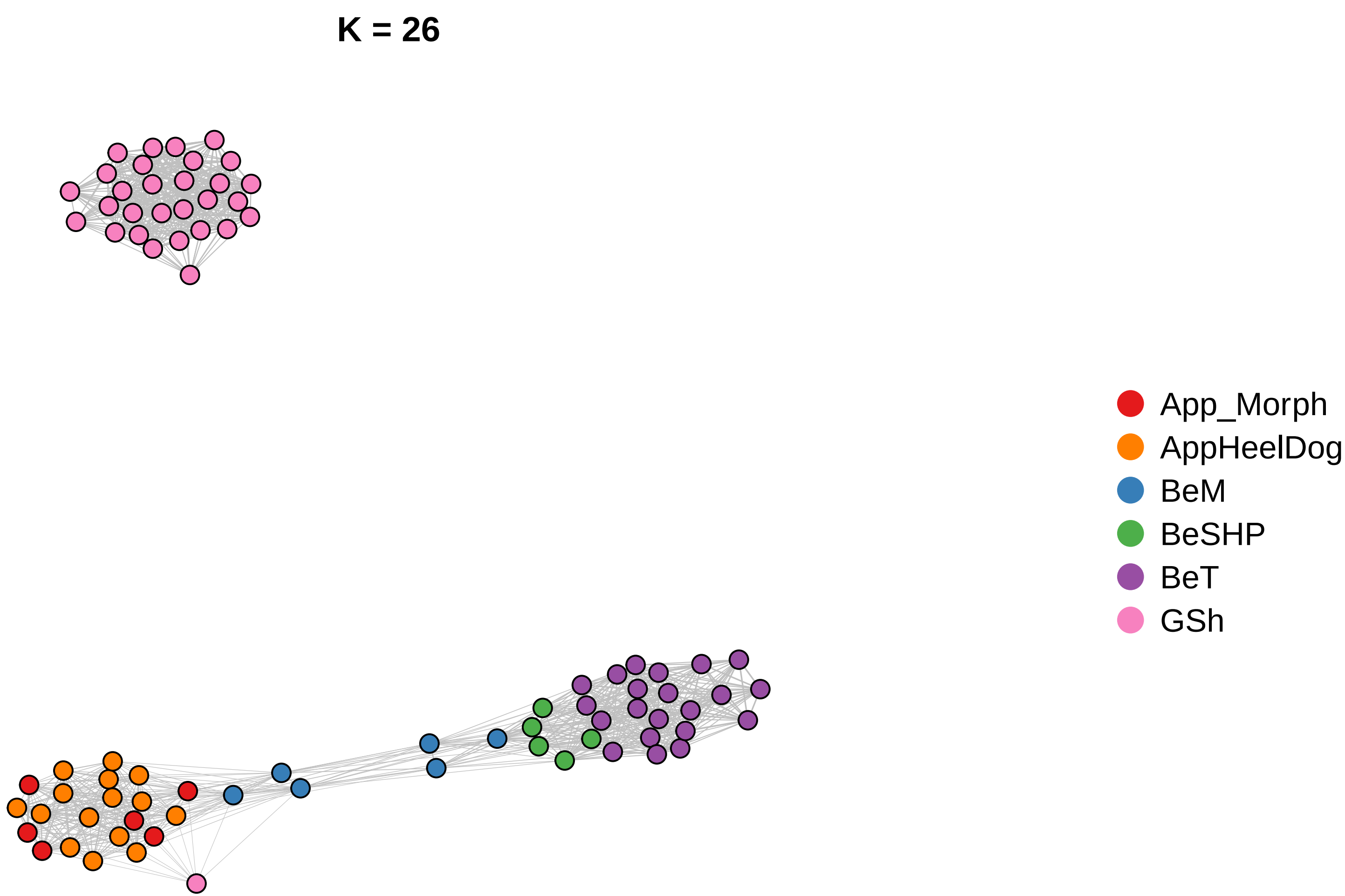
K = 26
App_Morph
AppHeelDog
BeM
BeSHP
BeT
GSh

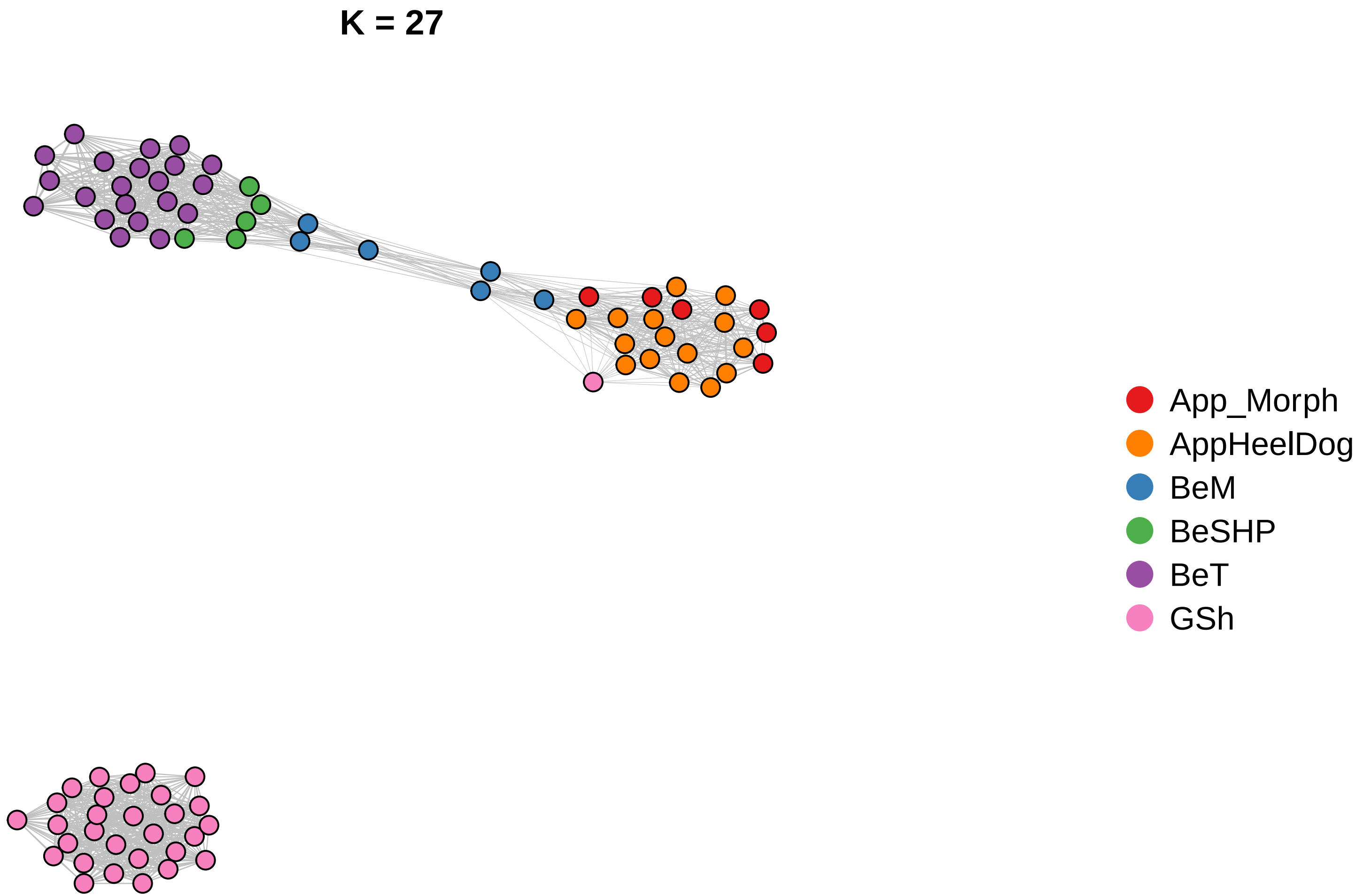
K = 27
App_Morph
AppHeelDog
BeM
BeSHP
BeT
GSh

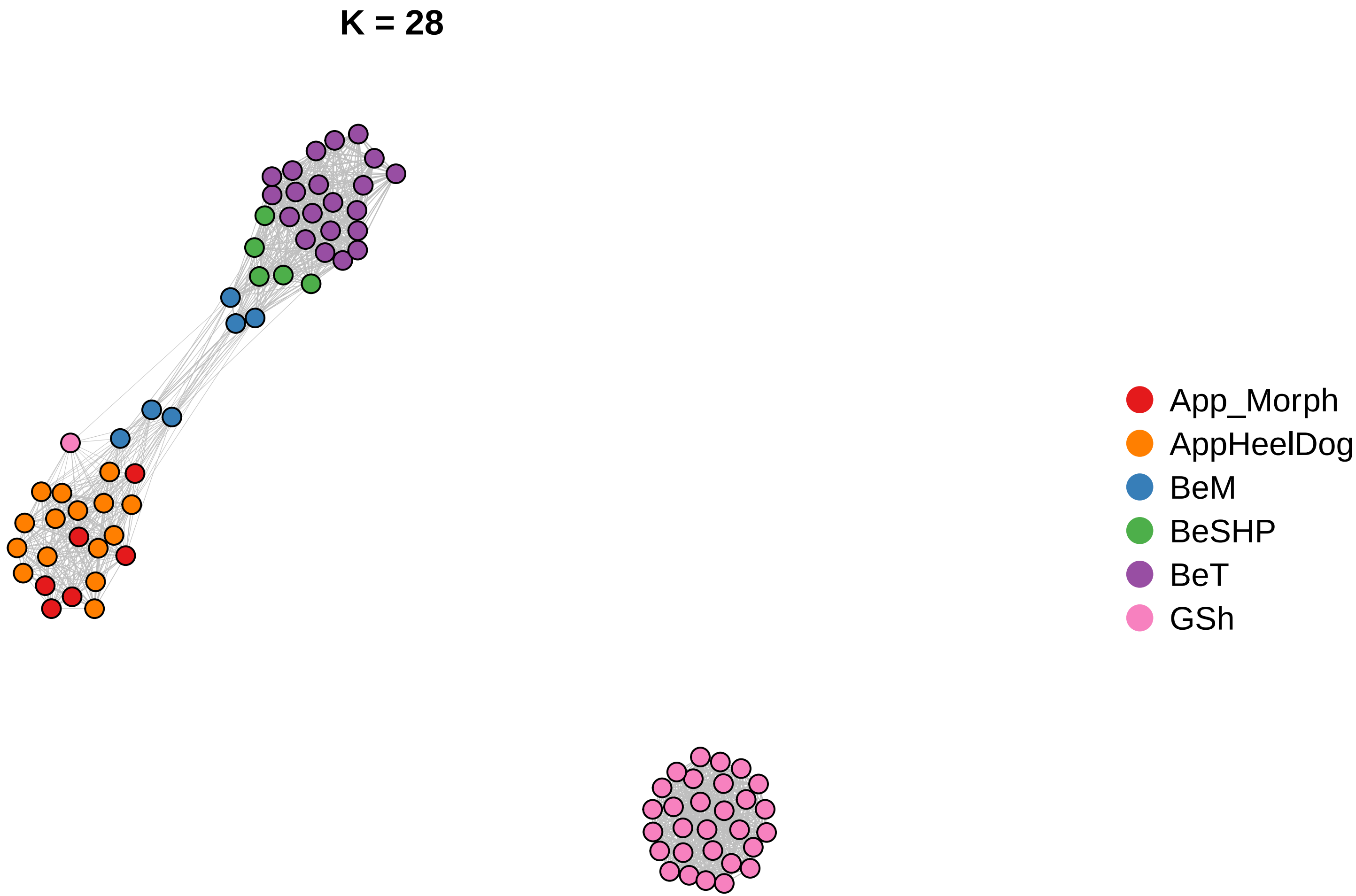
K = 28
App_Morph
AppHeelDog
BeM
BeSHP
BeT
GSh

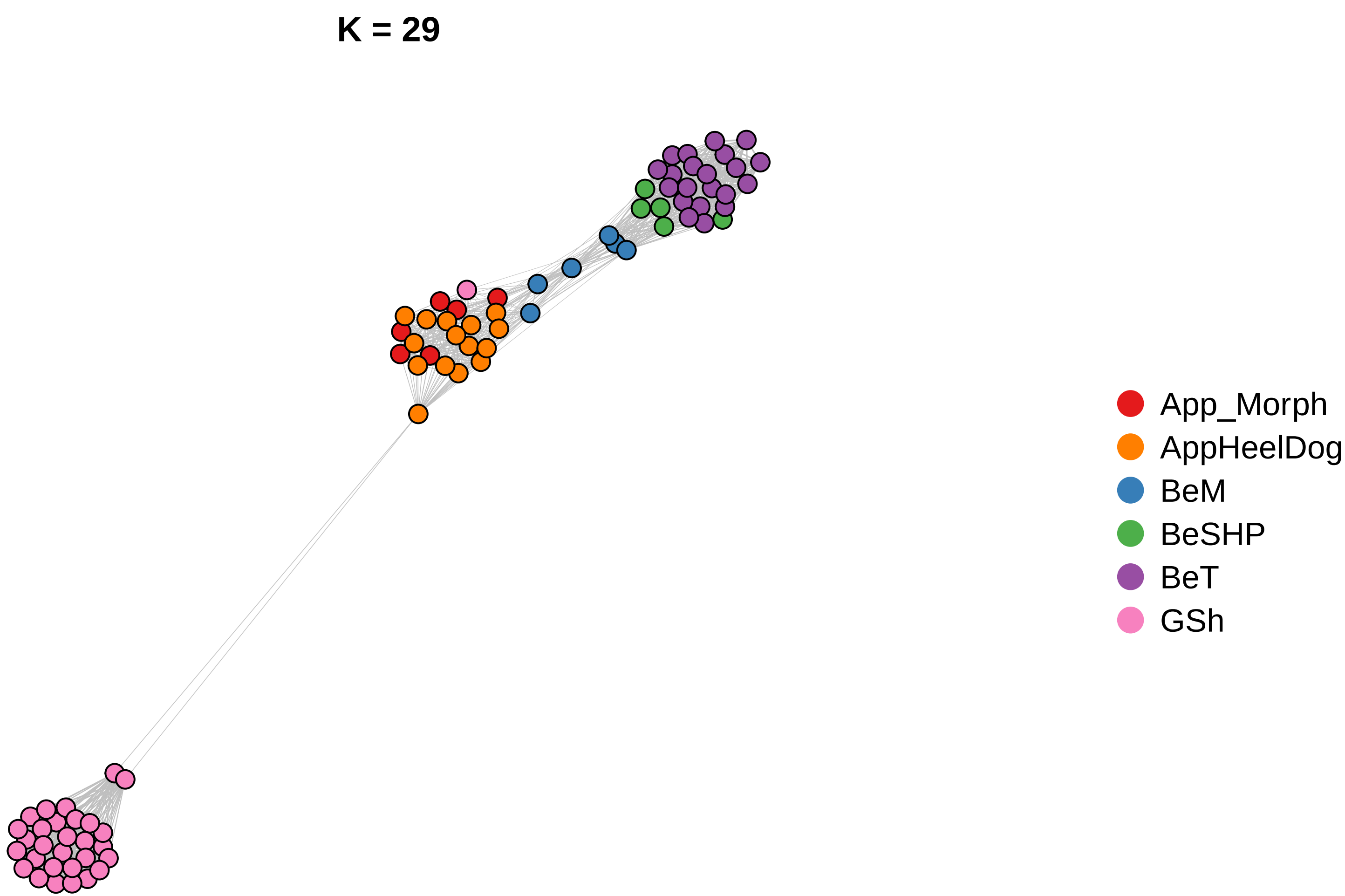
K = 29
App_Morph
AppHeelDog
BeM
BeSHP
BeT
GSh

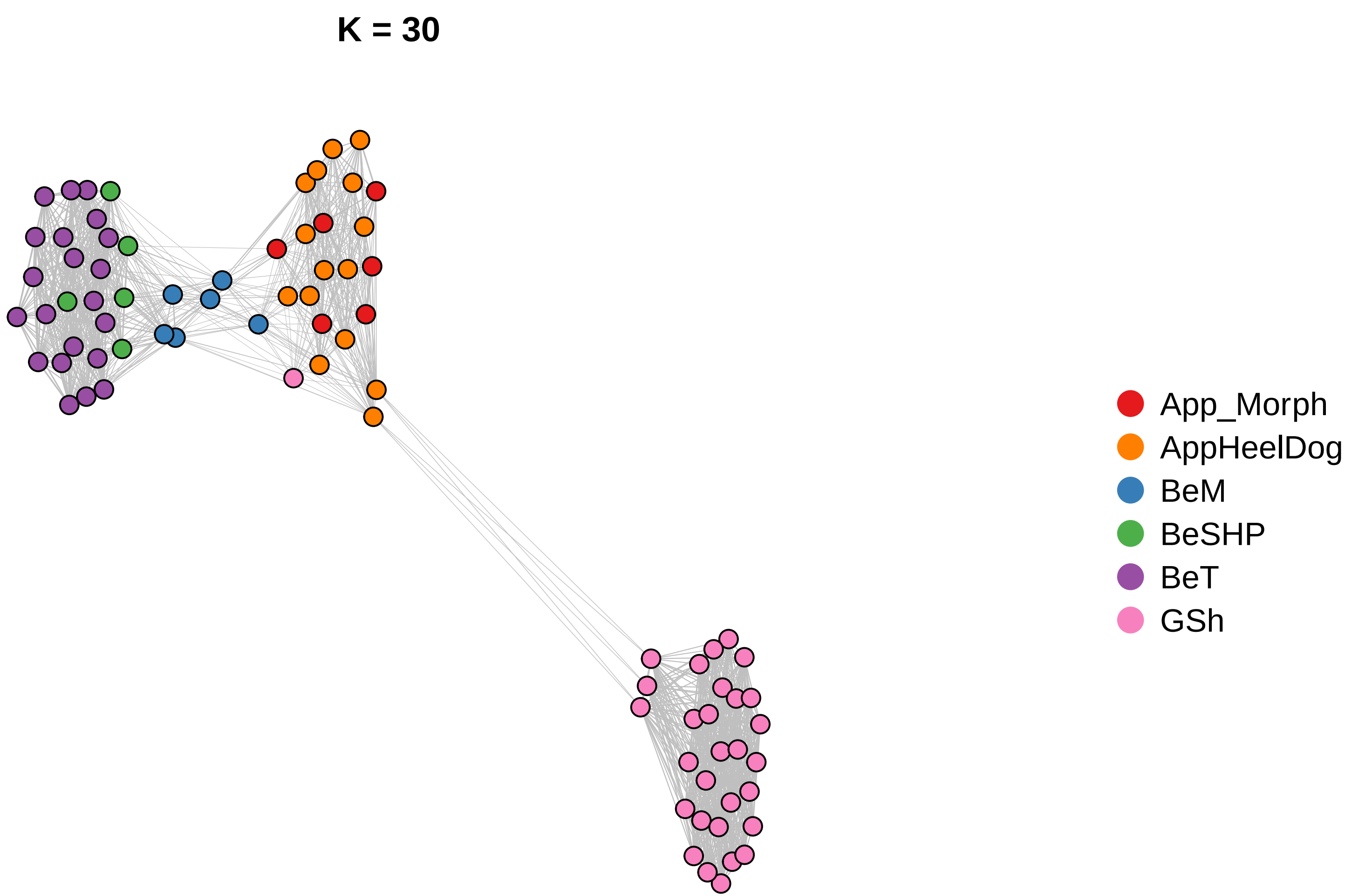
K = 30
App_Morph
AppHeelDog
BeM
BeSHP
BeT
GSh
